# Why we don’t want another “Synthesis”

**DOI:** 10.1186/s13062-017-0194-1

**Published:** 2017-10-02

**Authors:** Arlin Stoltzfus

**Affiliations:** 1 IBBR, 9600 Gudelsky Drive, Rockville, 20850 MD USA; 2 Office of Data and Informatics, National Institute of Standards and Technology, 100 Bureau Drive, Gaithersburg, 20899 MD USA

**Keywords:** Modern Synthesis, Evolutionary theory, Darwinism, Synthesis Historiography, Mutation-driven evolution

## Abstract

High-level debates in evolutionary biology often treat the Modern Synthesis as a framework of population genetics, or as an intellectual lineage with a changing distribution of beliefs. Unfortunately, these flexible notions, used to negotiate decades of innovations, are now thoroughly detached from their historical roots in the original Modern Synthesis (OMS), a falsifiable scientific theory. The OMS held that evolution can be adequately understood as a process of smooth adaptive change by shifting the frequencies of small-effect alleles at many loci simultaneously, without the direct involvement of new mutations. This *shifting gene frequencies* theory was designed to support a Darwinian view in which the course of evolution is governed by selection, and to exclude a mutation-driven view in which the timing and character of evolutionary change may reflect the timing and character of events of mutation. The OMS is not the foundation of current thinking, but a special case of a broader conception that includes (among other things) a mutation-driven view introduced by biochemists in the 1960s, and now widely invoked. This innovation is evident in mathematical models relating the rate of evolution directly to the rate of mutation, which emerged in 1969, and now represent a major branch of theory with many applications. In evo-devo, mutationist thinking is reflected by a concern for the “arrival of the fittest”. Though evolutionary biology is not governed by any master theory, and incorporates views excluded from the OMS, the recognition of these changes has been hindered by woolly conceptions of theories, and by historical accounts, common in the evolutionary literature, that misrepresent the disputes that defined the OMS.

**Reviewers:** This article was reviewed by W. Ford Doolittle, Eugene Koonin and J. Peter Gogarten.

**Electronic supplementary material:**

The online version of this article (doi:10.1186/s13062-017-0194-1) contains supplementary material, which is available to authorized users.

## Understanding the original Modern Synthesis

Soon after scientists, philosophers, and historians began discussing the status of the Modern Synthesis in the 1980s, it became clear that the Modern Synthesis conceived by scientists was not a fixed theory, but “a moving target” [1]. Scientists today may invoke it as an intellectual tradition defined by people and their ideas, or as a flexible framework that merely follows the implications of population genetics for evolution.

Actually, the architects of the Modern Synthesis— Mayr, Dobzhansky, Simpson, and others, drawing on earlier work by Fisher, Haldane, and Wright— attempted something far more ambitious. They proposed a coherent, falsifiable theory for how evolutionary genetics operates, claiming that it justifies a Darwinian view of evolution as smooth adaptation, renders all other modes of change either illegitimate or unnecessary, and provides a basis to unify evolutionary thinking across diverse fields such as paleontology, botany, zoology and genetics.

Understanding this theory— the original Modern Synthesis (OMS)— is vital to understanding, not just the past three decades of debate, but issues that have been debated for over a century.

The OMS emerged nearly seven decades ago, before we knew the detailed basis of any evolutionary change, and even before we knew that hereditary information is carried in chemical sequences. What the founders of the OMS knew— or thought they knew— was that, to account for evolution, the engine of adaptation must be powerful, and always ready. Given the choice of some possible modes of change, they favored the one that made adaptation rapid and powerful.

Thus, they appealed to the experimentally demonstrated way that selection can *create new types without mutation*, rapidly shifting the phenotypic mean of a population outside its original range by simultaneously shifting the frequencies of available alleles at many loci, leveraging recombination to combine many small effects in one direction. In Provine’s [2] seminal history of the foundations of the OMS, this is called the “effectiveness” or “efficacy” of natural selection, and scientists who accept it as the *sine qua non* of evolution are labeled as the proponents of Darwinism and selection.

Though this powerful mode of change by *shifting gene frequencies* depends on abundant pre-existing variation, it prevails in nature (the architects of the OMS argued) because natural populations have a “gene pool” in which recessivity and balancing selection (heterozygote advantage, negative frequency-dependent selection) *maintain* variation that is perpetually recombined (by sexual mixis, chromosome assortment, and crossing over). Events of mutation that introduce new alleles may occur, but play no direct role: evolution is initiated by a change in conditions that brings on selection, and recombination is the proximate source of the variation from which an adaptive response is shaped (see [3]; Additional file 1). All of evolution, including macroevolution, follows from *shifting gene frequencies*.

With this theory in mind, we can understand historic claims like that of Mayr (1963) [4] (for other examples, see Additional file 1):

It is most important to clear up first some misconceptions still held by a few, not familiar with modern genetics: (1) Evolution is *not* primarily a genetic event. Mutation merely supplies the gene pool with genetic variation; it is selection that induces evolutionary change. (p. 613)

In this way, the OMS invokes population genetics to justify a high-level view in which selection is a creative force that initiates and governs change, providing shape and direction, while variation is merely the source of fuel or raw materials— never a source of initiative, creativity, discontinuity, or direction, as it is in all non-Darwinian theories. The Darwinian view is distinctive in this dichotomy of explanatory roles, particularly the notion that selection is creative (e.g., p. 140 of [5]; [6, 7]). Selection is compared to a composer, sculptor, or painter, with variation supplying the notes, clay, or pigments [8]. Reviewing this position, Gould [8] concludes that “The essence of Darwinism lies in its claim that natural selection creates the fit. Variation is ubiquitous and random in direction. It supplies the raw material only. Natural selection directs the course of evolutionary change” (p. 44).

That is, the OMS is the synthesis of genetics and Darwinism, not the synthesis of genetics and selection accomplished earlier by the Mendelians. This may come as a surprise to readers familiar with the evolutionary literature, where the Mendelians are caricatured as “mutationists” who reject selection and smooth change, imagining evolution as merely a series of dramatic mutations (e.g., p. 305 of [9], p. 47 of [10], p. 67 of [11–14]). In reality, as historian Jean Gayon [15] explains, the Mendelians developed the modern concept of selection (p. 181 to 182), and “the fundamental doctrines of quantitative genetics were developed early in the century, long before the publication of Fisher’s canonical article of 1918 which is often credited with having laid the foundations of the discipline” (p. 316).

Mendelians such as Bateson, Morgan, Punnett, and others synthesized mutation, heredity and selection, laying the conceptual foundations for, among other things, the Hardy-Weinberg equilibrium, the biological species concept, the allelic selection model, and the multiple factor theory [16]. Although they imagined the possibility of smooth multifactorial change by selection, they did not insist that evolution is always Darwinian in behavior, but welcomed diverse ideas consistent with genetics, including selection as a stochastic sieve (acting on individual mutations), macromutations, one-step speciation, parallel evolution by parallel mutations, and tendencies due to biased variation [16].

The same features that distinguished the OMS from Mendelian-mutationism made it the kind of theory that could unify evolutionary biology. For the Mendelians (other than de Vries), there were no grand schemes or ruling principles: evolution was all about understanding the genetic details, e.g., characterizing the mutation spectrum. Their view provided little explanatory or predictive power, even for a geneticist— and few evolutionists were geneticists. By contrast, the OMS tells us that, once we understand the “gene pool” and the power of “shifting gene frequencies”, the genetic details cease to matter, and simple rules emerge. As Fisher (1930) [17] explains, the evolutionary researcher who understands this “will direct his inquiries confidently toward a study of the selective agencies at work throughout the life history of the group in their native habitats, rather than to speculations on the possible causes which influence their mutations” (p. 21).

Thus, when the acclaimed “population genetical approach” (p. 16 of [18]) of the OMS was applied to paleontology by Simpson, or to systematics by Mayr, this did not involve population genetics directly. Instead, they applied *verbal theories* blessed by Dobzhansky, Fisher, and Haldane: adaptation to changing conditions is likely because natural populations have abundant variation; variation-induced trends are impossible because mutation rates are too small; adaptation proceeds by infinitesimal shifts because this is theoretically most likely (and is supported by all the facts). These verbal theories are the arms and legs of the OMS, *the parts that do the work*. When Simpson applied the OMS to paleontology, he looked at an apparent trend in the fossil record, argued that it probably isn’t a trend, and concluded that, anyway, it must be gradual adaptation by selection because there is no alternative (e.g., p. 159 of [19]). When Mayr applied the OMS to systematics, he argued that speciation is due to gradual adaptation during periods of geographic separation.

## The disunification of evolutionary biology

The OMS failed rather quickly as a master theory when, in the early 1960s, the results of comparative sequencing prompted biochemists to invoke precisely the kind of mutation-driven view that Fisher and the architects of the OMS had sought to exclude.

In the OMS view, visible change is a smooth selection-driven shift in quantitative characters, and the underlying genetic change consists of simultaneous shifts in the frequencies of alleles at many variable loci. Comparisons of protein sequences revealed a long-term process that simply does not look like this. For each position compared between two species, each species typically has a specific amino acid that either matches or doesn’t match that of the other species, and the number of differences increases with the divergence time of the species. This suggested that evolution could be broken down into a series of individual amino acid replacements— a Markov chain of unit changes—, each reflecting a mutation that emerged at some point in time and rose to fixation. By contrast, the OMS says that where loci vary, each species will be defined by a different frequency distribution (i.e., of ancestral polymorphisms), and the process will be inherently multi-factorial— which is why Simpson (1964) [20] thought that making a phylogeny from just one locus (hemoglobin) was foolish, and “has nothing to tell us about affinities, or indeed tells us a lie.”

Though the results of sequence comparisons contradicted the OMS, the immediate effect of the molecular revolution was *not reform, but schism*: a new sub-discipline of “molecular evolution” emerged by 1971, with its own journals, meetings, and key theories— the molecular clock hypothesis and the Neutral Theory.

Molecular evolutionists celebrated the idea that “we need new rules in order to understand the pattern and dynamics of molecular evolution” [21]. The notion of a molecular-morphological “paradox” became a theme in the research literature [22]. King [23] interpreted the paradox to mean that all the conditions of the OMS— change is based entirely on pre-existing variation, no neutral alleles, no relation between rate of evolution and rate of mutation, and so on— apply at the morphological level, while exactly the opposite is true at the molecular level. For others, it meant that molecular evolution could be ignored as irrelevant to the classic questions covered by the OMS and all previous theories of evolution, e.g., Mayr proposed that molecular evolution provides only a superficial view of “proximate causes”, and later argued that fixation of neutral mutations is “not evolution”, and therefore, phrases such as “neutral evolution” or “non-Darwinian evolution” are illegitimate (p. 199 of [24]). In this way, a rather unequal truce was established.

These were the most visible signs of disintegration— the emergence of “molecular evolution” as a distinct scientific enterprise, the literal references to a “paradox”, and the organized rhetorical efforts of Mayr, Simpson and Dobzhansky to minimize the molecular threat to orthodoxy [22].

The deeper indications that the OMS had failed, and that evolution genuinely required new rules, appeared in mathematical theories. In particular, patterns of sequence divergence indicated the need for rules relating the rate of evolution directly to the rate of mutation, yet none existed [25, 26]. Mid-century mathematical population genetics, following the “shifting gene frequencies” theory, literally assumes that the alleles relevant to the outcome of evolutionary change are present initially, with no mutational introduction process, as others have noted [27, 28]. Because mutation rates in evolutionary models are often important only as rates of introduction, there are large bodies of classical theory that have no terms for mutation at all, e.g., none of the hundreds of equations in Edwards’s 1977 treatment of the mathematical foundations of population genetics [29] has a term for mutation (the word “mutation” appears only on p. 3 in the sentence “All genes will be assumed stable, and mutation will not be taken into account”).

In 1969, this gap was addressed (by King and Jukes, and by Kimura and Maruyama) with the origin-fixation formalism, which relates the rate of evolution directly to the rate of mutational introduction (“origin”) and to the probability of fixation [25]. Origin-fixation models are now a major branch of theory with many practical applications [25]. Early models used by molecular evolutionists typically invoked neutral or nearly neutral mutations. Subsequently, theoreticians interested in the genetics of adaptation began to explore new-mutations models, resulting in the minor renaissance described by Orr [30].

Thus, the development and use of mathematical models reveals unambiguously that the OMS does not suffice to depict evolutionary dynamics, because it fails to cover mutation-driven dynamics. This failure is not a mistake or oversight, but an intentional feature of the OMS reflected in the explicit claims of Mayr, Dobzhansky, Simpson, Stebbins and others that selection uses abundant variation in the “gene pool” and does not wait for new mutations (see [3], Additional file 1).

For this and other reasons, the OMS simply is not the foundation of contemporary thinking about evolutionary causes. The dynamics that give the “gene pool” its mojo are largely inapplicable in prokaryotes, *the organisms that have dominated the biosphere for most of its existence*. The OMS is not the theory used by molecular or microbial evolutionists (e.g., [31–33]), though it may be a perfectly good theory for special cases, e.g., for short-term changes in quantitative traits in large panmictic diploid sexual populations.

Of course, all such distinctions disappear if one chooses to equate all views of evolution that refer to genetics, but this equivalence was not assumed before. For instance, in historic sources from the 1930s to the 1970s, the two-step process by which a new mutation arises spontaneously, and then becomes established by virtue of an unsolicited advantage, is not called “adaptation” (which connotes a graduated response to a stimulus), but the “lucky mutant” view, “pre-adaptation”, or “random pre-adaptation” (p. 254 of [34], [35], p. 121 of [4], pp. 157, 236, 257 of [19], p. 325 of [36]). For instance, Simpson (1967) [19], referring to the Mendelians, complained that “the problem of adaptation was, in their opinion, solved by abolishing it: they proclaimed that there is no adaptation, only random pre-adaptation” (p. 276). The OMS deliberately rejects this mode of change as *a different theory for how evolution works*. Today lucky mutant models may be referenced as “Darwinian adaptation” or “Darwinian evolution” [30, 37], reflecting the way that mutationist thinking has been normalized, and even appropriated (see Additional file 1).

How do current conceptions of a “Modern Synthesis” or “Evolutionary Synthesis” relate to the OMS? The advocates of an “Extended Evolutionary Synthesis” (EES) [38, 39], as well as some opponents such as Lynch, see the standard orthodoxy as a commitment to explaining evolution in terms of population genetics. Yet, the OMS does not encompass the mutation-driven theories invoked by leading evolutionary geneticists such as Lynch [33], Lenski [40], Orr [41], or Nei [32], though all are consistent with population genetics.

In the *Oxford Encyclopedia of Evolutionary Biology*, the Modern Synthesis is defined as a flexible set of views that may absorb any challenge into a modified version of itself [42]. Many opponents of the EES, such as Coyne [43], Svensson [44], Welch [45] and Wray, et al. [46] rely on this kind of flexibility. Darwin’s repeated and explicit descriptions of the main causes of evolutionary modification in *The Origin of Species* do not invoke niche construction. Yet, Darwin worked on earthworms— a model system for niche construction— and noted how they modified their environment. On these grounds, Wray, et al., argue that a concern for niche construction is part of a mainstream tradition going back to Darwin, rendering an EES unnecessary. Svensson [44] likewise associates niche construction with a standard orthodoxy by associating Lewontin with it. The reasoning is not scientific, but cultural: it is about an intellectual tradition defined by people and their interests.

This kind of wishy-washy thinking, in which scientists ostensibly debating a scientific theory are actually aligning themselves with a cultural identity-group, can do no good. The word “theory” can have different meanings in science, but none of them would justify the Darwin-studied-earthworms-therefore-we-win argument.

## The corrupting influence of Synthesis historiography

The OMS was a bold conjecture that rejected the haphazard process of mutation and sorting envisioned by the Mendelians— mutation proposes, selection disposes (i.e., decides)—, redefining “evolution” as a powerful engine of adaptation that works by shifting frequencies of small-effect alleles at many loci simultaneously. The achievements claimed for the OMS (justifying Darwinism, refuting all rivals, and unifying the field) all depend on this “shifting gene frequencies” theory. Under its influence, generations of students were taught that evolution is “shifting gene frequencies.”

The words “shifting gene frequencies” are still taught today, but the meaning has been lost. How can it be that we forgot this historic theory, allegedly the centerpiece of 20^th^-century thinking?

Amnesia and distortion are perhaps inevitable given that scientists are regularly exposed to a Synthesis narrative, common in the evolutionary literature, in which a single Grand Unified Theory sweeps away foolish rivals, unites the field, and establishes a permanent orthodoxy. Without the constraints provided by crucial historical facts, conceptions of this grand theory have drifted, while the Synthesis story remains. What scientists need to understand is that this narrative was introduced, not by independent historical scholarship, but by the architects of the OMS themselves. Some historians call it “Synthesis Historiography” [47], i.e., telling history in ways that turn out right for the Modern Synthesis. Synthesis Historiography draws on stories in which opponents of Darwinism behave irrationally and hold views with obvious flaws, as in the Essentialism Story— “fabricated” by Mayr, according to historian Mary Winsor [48]—, the “eclipse” narrative, and the mutationism myth [16], so that the establishment of the Modern Synthesis appears as a victory of rationality and evidence over ignorance and superstition.

In short, the accounts of history in the evolutionary literature are not good sources for reliable information about developments in evolutionary thinking. In practice, they have fostered a kind of conceptual immune system: scientists guided by these stories have difficulty appreciating genuine alternatives, which are only known through caricatures, so that all reasonable ideas are assumed to align with the Synthesis. If evolution has some jumps or steps, this is literally interpreted as a moderate Darwinian position (e.g., [49]), contrary to the clear meaning of Darwin’s *natura non facit salta*, because saltationism is known only as a foolish extreme. If evolution is found to depend critically on the timing and character of individual mutations, this is conceptualized as a shift toward more “chance” or “contingency” within the Synthesis (e.g., [40]), credited to Gould rather than the Mendelians; it is not understood as a concession to innumerable historic critics who, for precisely this kind of reason, opposed the Darwinian conception of selection as a creative force; it does not lead to rejection of the obviously inadequate doctrine of raw materials, nor prompt a search for a better way to conceptualize the actual role of the introduction process in evolution.

Thus, conforming our understanding of evolutionary biology to fit the Synthesis narrative not only distorts history, but also distorts *science*: it results in a radical misappropriation of scientific credit, obscures useful distinctions that were clear to our intellectual progenitors, and encourages us to negotiate scientific novelty, not by conceptualizing and evaluating novel causal theories, but by patching up the old ruling principle of selection as “the ultimate source of explanation in biology” [50] with a set of vague non-causal concepts— “chance,” “constraints,” and “contingency”— to explain its stubborn refusal to rule.

Deference to the “Synthesis” is usually passive and unconscious, but sometimes it is a conscious decision, as in this defense offered by Futuyma [51]:

The seeming exclusivity of the ES [Evolutionary Synthesis] can be understood (and excused, if deemed necessary) only by appreciating the state of evolutionary discourse in the early twentieth century (see Simpson 1944; Rensch 1959; Bowler 1983; Reif et al. 2000). Darwinism was in “eclipse” (Huxley 1942; Bowler 1983), in that almost no biologists accepted natural selection as a significant agent of evolution. (The exceptions were chiefly some of the naturalists.)... Hugo de Vries and Thomas Hunt Morgan, founders of genetics, instead interpreted mutations as a sufficient cause of evolution... [omitted comments on Lamarckism and orthogenesis]... Those who today disparage the Evolutionary Synthesis as a constrained, dogmatic assertion that evolution consists only of natural selection on random genetic mutations within species must recognize that the authors of the Synthesis were responding to an almost complete repudiation of natural selection, adaptation, and coherent connection of macroevolution to these processes.

Indeed, “Darwinism” and “natural selection” were not highly regarded in the early 20^th^ century, when these terms denoted a refuted theory— Darwin’s non-Mendelian theory of “natural selection” by fluctuation, struggle and blending, refuted by Johannsen’s pure-line experiments [15, 52]. As Gayon [15] explains, “the decline of Darwinism was virtually always attributed to the experimental refutation of the hypothesis of ‘natural selection’ in the highly restrictive sense that Darwin had intended” (p. 2). In a less restrictive sense, the dichotomy of creative selection and inert raw materials (the potter and the clay) that lies at the heart of Darwin’s thinking [7] is widely questioned by well informed scientists today, not only in molecular evolution, but in evo-devo with its emphasis on “the arrival of the fittest” [53], a phrase popularized by de Vries himself [54].

To repeat the canard that “almost no biologists accepted selection as a significant agent”, and then to make it specific by naming de Vries and Morgan, indicates a stunning level of misplaced trust in Synthes is fables. Many books from that era are freely available in online facsimile editions, including the first textbook of genetics, by Punnett (1905) [55], which explains that mutations are heritable while fluctuations are not, concluding that “Evolution takes place through the action of selection on these mutations.” It is likewise easy to verify that de Vries [56] begins his major 1905 English treatise by writing that

Darwin discovered the great principle which rules the evolution of organisms. It is the principle of natural selection. It is the sifting out of all organisms of minor worth through the struggle for life. It is only a sieve, and not a force of nature... (p. 6)

and that Morgan [57], in the closing summary of his 1916 book, writes that:

Evolution has taken place by the incorporation into the race of those mutations that are beneficial to the life and reproduction of the organism (p. 194)

Having thoroughly misled the reader about history, Futuyma then declines to take the substantive claims of the OMS seriously, building his defense instead on personal sympathies. In the passage quoted above, he does not urge us to accept a theory on the grounds that the theory is correct, but orders critics to forgive the “authors of the Synthesis” for being dogmatic, because their opponents rejected selection, i.e., he appeals to identity-politics via nostalgia and falsehoods.

For each of the past five decades, evolutionary thinking has diverged further from the OMS, both into completely new territory, and into territory previously considered non-Darwinian. Advocates of the OMS used caricatures to poison sentiment against “mutationism”, yet since the 1960s, we have increasingly invoked mutation as a source of initiative, discontinuity, creativity and dynamics in ways that recall the thinking of the Mendelians [16]. Likewise, the word “saltation” remains radioactive, but the case for *natura non facit salta* was never solid [58], and today we know that evolutionary change includes discontinuities due to major-effect mutations. The architects of the OMS poured scorn on “orthogenesis”— at its best, simply a focus on developmental constraints [59]— but failed to establish their conjecture that selection is the sole source of direction in evolution and thus the ultimate source of explanation. Today the influence of mutational biases in diverse taxa is established (e.g., [60–62]), and the influence of developmental biases is under investigation (e.g., [63]).

The Synthesis narrative, maintained only by enormous sacrifices of rigor and clarity, must be abandoned. The OMS is a clever theory when considered as a special case, but proposing it as a master theory was premature, and claiming that it was established empirically was an exaggeration bordering on delusion. The fact that the OMS failed by the 1970s tells us two things: *the historical narrative of the Grand Unifying Theory is false*, and more importantly, *evolutionary biology does not need a master theory*.

The correct term to describe contemporary mainstream thinking in evolutionary biology is “contemporary mainstream thinking”. To call it an “Evolutionary Synthesis” or “Modern Synthesis” shows a disregard for scholarly rigor. There is no flexible “Synthesis”, but rather (1) a scientific discipline that changes its views appropriately, based on the latest findings, and (2) conformists spinning out increasingly flimsy versions of the claim that evolutionary biology is governed by a flexible master theory that traces back to Darwin through Mayr, et al.

## The importance of genuine scientific theories

Prior to the “Synthesis”, most evolutionary biologists invoked multiple means or modes of evolutionary modification. For Darwin, the 3 primary means of modification were natural selection, use-and-disuse (Lamarckian modification), and direct inherited effects of the environment.

Separating the totality of evolutionary change into different aspects or modes, each of which follows distinct rules and implicates causal factors differently, is a valuable goal for evolutionary theorizing. For instance, today we recognize a mode of neutral evolution that, under certain conditions, follows a particular set of rules, but is not assumed to be universal.

In the past, another major goal of evolutionary theorizing was to develop a universal theory that subsumes all evolutionary behavior. The grand project undertaken by the architects of the OMS was based on the bold claim of universality from the opening chapter of Fisher, 1930 [17]: once one accepts that evolution has a Mendelian basis, Darwinism follows, and all other views must be set aside. This theory claimed to be, not just universal in the sense of covering everything (at least, everything important), but unified and cohesive in the sense of covering everything with one mode of evolution— smooth adaptive shifts via shifting gene frequencies.

Subsequently, this failed conjecture has been substituted with a *different claim of universality* to the effect that evolutionary causation is adequately described in terms of population genetics. This is a reasonable suggestion, but it is a different kind of claim with a different history. There is nothing Darwinian about it. Associating it distinctively with the “Synthesis” would be problematic. For instance, when Bateson and Saunders (1902, p. 130) [64] give a perfect verbal rendition of the Hardy-Weinberg equilibrium and its use in detecting “disturbing factors”, they are clearly appealing to what became known as “population genetics”, decades before Dobzhansky (1937) [65] declared that “the mechanisms of evolution constitute problems of population genetics” (p. 11). Most importantly, to propose that evolution can be, or must be, understood via population genetics is a curiously empty “theory” relative to historic attempts to specify the nature and importance of the high-level causes of evolution, e.g., the origin of novelty, internal vs. external sources of direction, etc. This “theory” does not appear to be a falsifiable conjecture, but is more like a methodological claim about the most productive way to think about evolutionary problems. To invoke this as though it were a master theory is to confess that there is no such thing.

Indeed, abandoning the notion of a unified master theory is an obvious reform for 21^st^-century evolutionary biology. When the implicit demand for such a theory is removed from the current EES debate, for instance, what is left is a set of causal factors relevant to niche construction, developmental bias, and phenotypic plasticity, each of which deserves to be evaluated on its merits. Debates over such factors would be more productive if proponents of novel causes were to follow the model of Kimura’s Neutral Theory, which does not merely invoke a possible mode of change, but makes a precise general claim about the size of its effects in evolution.

This kind of conceptual reform is possible without revolution. Evolutionary biology was changed permanently by the critique of “good for the species” arguments by Williams [66], and by the take-down of naive adaptationism by Gould and Lewontin [67]. These reformers subverted conventional habits of thought by exposing their shallowness. Today, wishy-washy defenses of an ongoing “Synthesis” are easy targets for a badly needed reform in our ongoing discourse on the state of evolutionary thought: rejecting Synthesis propaganda, and accepting evolutionary biology as a legitimate scientific discipline that entertains bold conjectures about the measurable effects of novel causes, with no need for a master theory. The era of master theories based on ruling principles and grand schemes is long past. The OMS was the last such theory. There will not be another.

## Reviewers’ comments

### Reviewer’s report 1: W. Ford Doolittle

Stoltzfus boldly wades into the muddy waters of the Modern Synthesis and recently proposed “extended” versions thereof, and concludes that we don’t need any such grand theorizing. There are many points he makes that I like, and since this is a topic he knows better than I, I cannot fault his history other than in a general way. Each of us has come to believe what we do about evolution through idiosyncratic combinations of reading, listening and thinking, and I doubt that there is any one true story about this. Certainly evolutionary biologists suffer from the fact that our major or most widely read history writers have generally been practitioners with their own axes to grind. We would not expect politicians to write unbiased political histories, but we biologists seem to trust our own kind, as if scientists can more easily transcend themselves. My generation of molecular biologists (or at least I) took far too long to realize that Ernst Mayr was not in fact a disinterested observer. Stoltzfus points this out, and shows how the OMS (the original modern synthesis) assumed that relevant populations already harbor sufficient relevant genetic variability to adapt to most environmental challenges.

Author response: *One must agree with Doolittle that histories are written by people and not disinterested machines. We could respond to this by insisting that authors disclose their allegiances and hidden agendas, as he suggests below. Yet, science is also written by people, and we do not require authors to disclose their sympathies for various theories or research traditions, as if scientific papers were impossible to evaluate without a biography of the authors. Instead, we read scientific papers with an attitude of skepticism, and an openness to reason and evidence. One must approach accounts of history in the same way, which is why the arguments in the main text are built on citations and verifiable claims (to spare the reader some of the effort required for verification, quotations are provided in Additional file* 1). *This may be contrasted with story-telling that lacks verifiable claims based on named sources, forcing the reader to rely on trust alone.*

With the OMS, there was little need to worry about where that allelic variation came from and whether or not it was “random”. It is in a sense proof of this contention that any mutational or development non-randomness is generally seen as a “constraint” on proper evolutionary processes rather than their source.

Author response: *As Doolittle points out, the language of “constraint” or limitation refers to the case of unconstrained or unlimited evolution. Presumably, our understanding would be improved by conceptualizing evolution in terms of causal factors with positive effects, rather than invoking explanatory concepts to account for deviations from a counterfactual ideal that has never been clearly articulated.*

I think that Stoltzfus is also right that molecular evolutionists from the outset had a different mindset, focusing on lineages of amino acid replacements. They were also, which Stoltzfus does not point out, often from a different community and differently trained. Molecular evolutionary methods have now come to dominate even traditional morphology-focused subdisciplines of metazoan systematics, but in the beginning their application to prokaryotes brought in molecular biologists who neither knew nor cared much about the OMS.

Author response: *As Doolittle suggests, the influence of early molecular evolutionists, whose training differed from that of mainstream evolutionary biologists, deserves more attention. Starting in the late 1950s, Anfinsen (trained as a biochemist), Dayhoff (computational chemist), Zuckerkandl (physiologist with molecular focus), Jukes (biochemist), Fitch (biochemist) and others introduced new ways of conceptualizing evolution and analyzing its patterns, e.g., Dayhoff’s fully empirical and quantitative model of transitions between discrete amino-acid states, or Anfinsen’s methods of functional inference from patterns of dispensability and conservation (e.g., Ch. 6 of [* 68 *]). Rather than being incorporated directly into mainstream evolutionary thought, these new ideas were channeled into the upstart discipline of molecular evolution, which was separate for decades. By the time the two streams merged, the molecular stream was the more powerful, fed by enormous amounts of systematic data analyzed by powerful algorithms, thus the influence of the above molecular pioneers— still unacknowledged— is now enormous.*

Stoltzfus is right, too, I think, in holding that efforts to extend the modern synthesis (or conservatively to hold that such extensions were already prefigured in Darwin’s writings) “is not scientific, but cultural: it is about intellectual tradition defined by people and their interests.” Few of us want to discredit Darwin: we merely seek to show that our predecessors have interpreted him wrongly. I do think some of the language here is unduly harsh, condemning previous recountings of OMS history as “wishy-washy” or “nostalgia and falsehoods”. Stoltzfus too is writing history to emphasize the unfairness with which his own special interest (mutation) has been neglected. This is part of the genre, and and maybe we cannot eradicate it, but we can acknowledge that we are not disinterested.

Stoltzfus concludes that … “The era of master theories based on ruling principle and grand schemes is long past. The OMS [Original Modern Synthesis] was the last such theory. There will not be another.” I hope he’s right. That’s what should happen. But as long as biologists with any rhetorical flair feel obliged to reconstruct disciplinary histories so as to make their own views seem novel but consistent with what Darwin –if he knew what we know now – would think, such metanarratives will probably not go away. High-profile journals and the popular press seem to demand them, and they are what we often seek to defend from anti-evolutionists, who continue to search out the gaps between our circled wagons.

The tack I and colleagues take (Booth, Mariscal and Doolittle 2017, Ann Rev Micro 70:279-297), in reviewing the impact of microbial genomics on the Modern Synthesis is to see the latter as a useful aid in persuading biologists that nothing in their discipline makes sense except in the light of evolution. But that goal was achieved long ago, and the aid has became an impediment. The only metanarrative that we need to buy into now is that contemporary biological adaptation and diversity can in principle be explained by the interplay of biological and abiotic processes that we (mostly) understand, operating over about 4 billion years. We suggest that evolutionary biological theory should be recast as a “historically and loosely connected toolkit of concepts, methods, models and mechanisms, concatenations of which can explain how individual changes might have been effected in individual molecules, organisms, or lineages …”. It is my understanding that modern historians have mostly adopted such a limiting, piecemeal, toolkit perspective. What is evolutionary biology, after all, but history without (or less of) a focus on culture?

Author response: *Doolittle raises several important points here. Scientists routinely use the word “theory” for two different concepts, one of which is the set of abstract principles relevant to some topic, as in “music theory” or “population genetics theory”, and the other of which is a grand hypothesis, a conjecture subject to empirical evaluation, as in Kimura’s “Neutral Theory of Molecular Evolution” or Gilbert’s “Exon Theory of Genes”. We can distinguish these as theory*_*A*_ *(abstract, analytical) and theory*_*C*_ *(concrete, conjectural). This distinction (explained in more detail in [* 69 *]) is not a complete description of how the word “theory” is used, but it is a good start. For instance, by applying this distinction, we can understand Kreitman’s article “The Neutral Theory is Dead. Long live the Neutral Theory.” [* 70 *] as an argument to reject the Neutral Theory*_*C*_ *while retaining neutral null models (neutral theory*_*A*_ *) for hypothesis-testing.*

*The reference by Booth, et al to a “toolkit” sounds like a reference to evolutionary theory*_*A*_, *which consists of all the reliable principles of reasoning available to evolutionists, including various models, algorithms and equations. If we limit theory*_*A*_ *to formalisms whose validity can be evaluated on logical principles, there is not much room for disagreement about what is included.*

*Instead, the disagreements are about theories*_*C*_. *Evolutionary biology seems to be unique in this regard. One does not talk about “the theory*_*C*_ *of chemistry” or “the theory*_*C*_ *of economics” because, in other fields, there is no presumption of a master theory*_*C*_. *Today, the expectation of a master theory*_*C*_ *of evolution, it seems to me, comes largely from outside the scientific research enterprise, from popularizers and culture warriors, which is perhaps what Doolittle means to suggest. However, the mid-century Synthesis movement clearly claimed to have a theory*_*C*_ *that accounts generally for evolution.*

*As to the views of historians on this theory, the late William Provine (1971) [* 2 *] identifies its foundation with acceptance of the power of shifting frequencies (of small-effect alleles in the gene pool) to create new types without mutation, inspired by Castle’s experiments with the hooded rat. Provine said later [* 71 *] that the Modern Synthesis “came unraveled” in the 1980s, calling the gene pool “one of the most artificial concepts of population genetics” and pointing out the inadequacy of assuming that recombination (rather than mutation) is the proximate source of variation, and of assuming that macroevolution is a simple extension of shifting gene frequencies. That is, Provine identifies a theory*_*C*_ *that aligns with what is here called the OMS, and claims that it failed for some of the same reasons we would say that the OMS has failed.*

*By contrast, according to Smocovitis [* 1 *], historians gave up on trying to define the Modern Synthesis as a theory*_*C*_, *e.g., she writes that*

*“by the late 1980s the notoriety of the evolutionary synthesis was recognized... So notorious did ’the synthesis’ become, that few serious historically minded analysts would touch the subject, let alone know where to begin to sort through the interpretive mess left behind by the numerous critics and commentators” (p 43).*

*The difference is that Provine became an expert in theoretical population genetics so that he could make his own determination of history from reading the scientific literature, doing so in the 1960s— when confidence in the Modern Synthesis was high—, whereas Smocovitis and others came along in the 1980s, when the OMS had failed and revisions were demanded, relied on scientists to explain the significance of the Modern Synthesis, and reached the conclusion that it is a “moving target”.*

*Doolittle also draws attention to rhetorical strategies used to negotiate the threat posed by unfamiliar ideas. Sometimes reformers attempt to appropriate Darwin’s name to gain sympathy with fans, and in other cases, conformists attempt to appropriate new developments for Darwin, to foster the impression that no reform is needed. In this way, Darwin is credited with all manner of proposals, by first broadening the proposal to make it an easy target, and then searching for a scrap of textual evidence in the works of Darwin (but not of other early thinkers). The earthworm argument by Wray, et al. is a recent example. Apparently Wray, et al. have no strong scientific disagreement with their reformist opponents about niche construction, but merely wish to appropriate niche construction for the Darwin brand. Perhaps “earthworming” would be a good name for this gambit, e.g., Lynch (2007) [* 72 *] earthworms the proposed role of mutational and developmental biases in the introduction of variation [* 73 *] by mislabeling it as “mutation pressure”, asserting it is not new, then citing Darwin and a string of others with no clear connection to the actual proposal.*

### Reviewer’s report 2: Eugene Koonin

In his Opinion article, provocatively titled “Why we don’t want another “Synthesis””, Arlin Stoltzfus combines a brief historical discussion of the Original Modern Synthesis (OMS) of evolutionary biology with a critique of ideas on the “extended synthesis” and a declaration that our view of the very character of evolutionary biology should change. More specifically, the author posits that there can be no “master theory” of biological evolution: the OMS was the last attempt that failed, and a new one is neither feasible nor desirable. The article is interesting, thought-provoking and very well written, so I think it will be of interest, above all, to any evolutionary biologist, but potentially, to many other biologists as well. I do not think I have strenuous objections to anything the author has to say. However, I do believe that some of the statements in the article can be misinterpreted, and I hold a mildly dissenting position that is outlined below.

The author clearly recognizes the shortcomings of OMS as the core theory of evolutionary biology. The main omission of OMS is proposed to be mutation-driven evolution but it is also pointed out that “The dynamics that give the “gene pool” its mojo are largely inapplicable in prokaryotes, the organisms that have dominated the biosphere for most of its existence. The OMS is not the theory used by molecular and microbial evolutionists (e.g., [31–33])...” What the author does not point out, is that evolution of prokaryotes (and to a lesser extent, eukaryotes, especially unicellular forms) involves key processes, such as horizontal gene transfer, that are distinct from typical mutations, and furthermore, fly in the face of gradualism. Furthermore, I would submit that it is becoming clear that the entire course of evolution of prokaryotes, but to a large extent, eukaryotes as well is, to a large extent, shaped by the coevolution of these organisms with genetic parasites including viruses, transposons, and more. This notion is orthogonal to the OMS, even when supplemented with the concept of mutation-driven evolution, it is simply not part of what is perceived “fundamentals of evolutionary biology”. I think that these major new phenomena that were either plainly unknown to the architects of the OMS (and even the leader of a later era in evolutionary biology, such as Kimura) or not adequately appreciated at the time, call for some new theoretical frameworks. Quite a few attempts in these directions have been published (not citing here, easy to find).

Author response: *Koonin is right to point out that phenomena other than mutation-limited evolution could be invoked as innovations not anticipated in the OMS, including the role of molecular macromutations, clearly inconsistent with gradualism, and with the doctrine that selection rather than mutation is creative. The focus on mutation-limited dynamics in my article should not be taken to imply that this is the only deficiency, nor even “the main” deficiency. Instead, it is the most poignant deficiency for the present purposes: it is a case of rejection rather than omission, where the historical record of this rejection is clear, and the inadequacy of the OMS position is clear. For such reasons, this particular deficiency reveals most clearly the actual historic importance of a genuine OMS theory*_*C*_ *that (like most such theories) takes risks in the service of a simplifying ideology.*

I tend to agree with the author that a single, coherent master theory of biological evolution is likely to be a pointless pipe dream (although I am perhaps less certain). However, this does not imply that conceptual generalizations incorporating new discoveries are not desirable or even necessary for further progress of research in evolutionary biology. In my view, these should take (and, actually, I think are taking) the form of a network of multiple formal theoretical models joined by less formal concepts (this view is explicated in Ref. 31 of Stoltzfus’s paper, and do not see why it might become irrelevant). To me, this type of association of theoretical concepts is most naturally described with the term Synthesis. I do realize that the OMS was (and perhaps, is, at least, by some) perceived as something quite different, namely a single “master theory”. I nevertheless wonder whether the title and some of the discussion in Stoltzfus’s article might be inadvertently misleading. It perhaps might make sense to make some amendments, in order to present a more balanced outline of what kind of conceptual developments in today’s evolutionary biology are feasible and useful, and which are not.

Author response: *This point can be clarified as follows. The argument is not against synthetic thinking generally. The “Synthesis” movement of the mid-20* ^*th*^ *century was a specific historical event that established a cultural identity for evolutionary biology tied to a master theory of evolution (the OMS), a set of canonical founders (Mayr, et al), and an interpretation of intellectual history. Generations of students learned that the founding of the discipline was a victory of reason over unreason that restored Darwin’s thinking and ended a dark period called the “Eclipse”. Many scientists identify with this tradition and feel bound to defend it, with the inevitable result of distorting science, as we can serve but one master.*

*We don’t want another “Synthesis” in the sense of a deliberate campaign to establish a cultural identity that, in the future, will be protected jealously by conformists.*

*In addition, I suggest, we don’t want another master theory, for reasons Koonin surely appreciates already. In evolutionary biology, we want to understand the evolution of animal body plans on the time-scale of hundreds of millions of years. We also want to understand— so as to develop more effective drug therapies— the progression of an HIV infection in a single patient over the course of months, with the virus population evolving and the patient’s immune system responding, under various treatment regimes. We also want to understand the evolution of genome content in prokaryotic species in which two typical members share only 60% of their genes, and the remainder is conceptualized as part of a metagenome distributed and shared among an unknown number of other species. A cohesive master theory with the generality to cover just these 3 cases would have to be something relatively empty, and discussing relatively empty theories is not a good use of our time.*

### Reviewer’s report 3: J. Peter Gogarten

Arlin Stoltzfus’s article is an interesting read that provides a breath of fresh air to the debates on the modern synthesis. The manuscript considers that a multitude of processes occur in evolution, and that the extent to which these processes are applicable differs for different groups. Stoltzfus’s description of the historical and ego centered controversies and the impact these had on getting new ideas accepted should be a warning to evolutionary biologists, help us to keep an open mind towards the many processes that occurred and occur in evolution, illustrate that in teaching and writing we should be careful with simplifying solutions to complex problems, follow both sides of an argument and be aware that one side’s description of the other side’s opinion can be rather biased. One possible improvement would be to make the abstract a little easier to comprehend; however, as a Ford Doolittle once remarked to me, it is ok to present a complex thought in a complex way, then the reader has to struggle to follow the argument and ends up owning it.

Author response: *The abstract has been rewritten for improved clarity.*

## Additional file

Additional file 1 Supporting Quotations. (PDF 114 kb)

## Abbreviations

EES: Extended evolutionary synthesis
OMS: Original modern synthesis
