# Supplementary material for: Why we don’t want another “Synthesis”
Source: Biol Direct. 2017 Oct 2;12:23. doi: 10.1186/s13062-017-0194-1 (PMC5625744; doi:10.1186/s13062-017-0194-1)
Supplement: Additional file 1 — Supporting Quotations. (PDF 114 kb) [file 13062_2017_194_MOESM1_ESM.pdf]

OPINION

Supporting Quotations

Supplementary material for ”Why we don’t want another ’Synthesis’”

Arlin Stoltzfus

Correspondence: arlin@umd.edu
IBBR, 9600 Gudelsky Drive, 20850
Rockville, MD, USA
Full list of author information is available at the end of the article

The main text makes several claims about the OMS and the preceding views of the Mendelians that are not well known among scientists, or that contradict what one finds commonly in the evolutionary literature. Nevertheless, these claims are readily documented from primary sources. For documentation of the ideas of the Mendelians, the reader is referred to Stoltzfus and Cable (2014) [1]. The quotations included below focus on (1) OMS theories of how population genetics works in nature, (2) contemporary views that contradict the OMS in regard to the role of mutations and mutation rates, and (3) contemporary statements suggesting that the OMS has been forgotten, and that mutationist thinking has been normalized.

The Modern Synthesis is universally associated with mid-century books by a handful of ”founders” or ”architects”, the most oft-cited being Mayr, Dobzhansky, Simpson, and Stebbins, followed by Huxley, Ford and others. For the present purposes, it is appropriate to focus on the works of these authors in the period after they converged on a cohesive theory of evolutionary genetics, and began using common language to describe its distinctive features. Below are some of their statements about mutation that invoke specific theories of population genetics.

> ”It is most important to clear up first some misconceptions still held by a few, not familiar with modern genetics: (1) Evolution is not primarily a genetic event. Mutation merely supplies the gene pool with genetic variation; it is selection that induces evolutionary change.” (p. 613 of Mayr (1963) [2])

> ”The study of evolutionary rates is a branch of the science of evolution that has been undeservedly neglected. One solution, though frequently proposed, is almost certainly wrong: that rates of evolution are controlled by rates of mutation. Indeed, there may not be any correlation whatsoever between the two phenomena.” (p. 7 of Mayr, 1959 [3])

> ”Those authors who thought that mutations alone supplied the variability on which selection can act, often called natural selection a chance theory. They said that evolution had to wait for the lucky accident of a favorable mutation before natural selection could become active. This is now known to be completely wrong. Recombination provides in every generation abundant variation on which the selection of the relatively better adapted members of a population can work.” (p. 38 of Mayr (1994) [4])

> ”Our calculations lead us, therefore, to the conclusion which has been reached by most geneticists who are studying evolutionary processes. The chief limiting factor on the supply of variability for the action of natural selection is not the

> availability or rate of occurrence of mutations, but the restrictions on gene exchange and recombination which are imposed by the mating structure of populations and the structural patterns of chromosomes. Natural selection directs evolution not by accepting or rejecting mutations as they occur, but by sorting new adaptive combinations out of a gene pool of variability which has been built up through the combined action of mutation, gene recombination, and selection over many generations." (p. 30-31 of Stebbins (1966) [5])

> "mutations are rarely if ever the direct source of variation upon which evolutionary change is based. Instead, they replenish the supply of variability in the gene pool which is constantly being reduced by selective elimination of unfavorable variants. Because in any one generation the amount of variation contributed to a population by mutation is tiny compared to that brought about by recombination of pre-existing genetic differences, even a doubling or trebling of the mutation rate will have very little effect upon the amount of genetic variability available to the action of natural selection. Consequently, we should not expect to find any relationship between rate of mutation and rate of evolution. There is no evidence that such a relationship exists." (p. 29 of Stebbins (1966) [5])

> "Novelty does not arise because of unique mutations or other genetic changes that appear spontaneously and randomly in populations, regardless of their environment. Selection pressure for it is generated by the appearance of novel challenges presented by the environment and by the ability of certain populations to meet such challenges." (p. 160 of Stebbins (1982) [6])

> "The process of mutation supplies the raw materials of evolution, but the tempo of evolution is determined at the populational levels, by natural selection in conjunction with the ecology and the reproductive biology of the group of organisms" (p. 282 of Dobzhansky (1955) [7])

> "Another misjudgment is to suppose that either the kind or the speed of evolutionary changes has been determined by how often the mutations occurred. Although repeatedly shown to be mistaken, this misconception recurs again and again, even in recent literature. The mutation process is, however, not synonymous with evolution; it is only the source of raw materials for evolutionary changes. " (p. 315 of Dobzhansky (1974) [8])

> "The large number of variants arising in each generation by mutation represents only a small fraction of the total amount of genetic variability present in natural populations. . . . It follows that rates of evolution are not likely to be closely correlated with rates of mutation . . . Even if mutation rates would increase by a factor of 10, newly induced mutations would represent only a very small fraction of the variation present at any one time in populations of outcrossing, sexually reproducing organisms." (p. 72 of Dobzhansky, et al. (1977) [9])

An important feature of OMS advocacy, evident in the above passages, is that speculative claims are advanced with deceptive confidence. The reader of Stebbins's claim that "mutations are rarely if ever the direct source of variation" will

assume that this generalization is based on knowledge of the detailed basis of many evolutionary changes, yet no such data were available. Dobzhansky's claim that a relationship between the rate of evolution and the rate of mutation has been "repeatedly shown to be mistaken" appears to be a case of a very solid claim, but it is not. Of course, these statements were not intended to deceive: the architects of the OMS were confident that they understood how evolutionary genetics operates in nature, and they aimed to persuade readers to adopt their theory and to reject alternatives.

As a rationalization of Darwinism, the OMS appeals to population genetics to argue that the course of evolution is directed by selection and not by variation, which merely supplies raw materials. The passages below reveal some aspects of this rationalization. The background for the statements of Huxley and Ford is the opposing pressures argument of Fisher and Haldane (explained in [10]) to the effect that the trend of evolution must be due to selection because mutation rates are small (see also p. 509 of [11]).

> "For no rate of hereditary change hitherto observed in nature would have any evolutionary effect in the teeth of even the slightest degree of adverse selection. Either mutation-rates many times higher than any as yet detected must be sometimes operative, or else the observed results [possible trends in the fossil record] can be far better accounted for by selection." (p. 56 of Huxley, 1942 [11])

> "An evolutionary line of organisms which is changing through eons of time can be likened to an automobile being driven along the highway. Mutation then corresponds to the gasoline in the tank. Since it is the only possible source of new genetic variation, it is essential for continued progress, but it is not the immediate source of motive power. This source is genetic recombination, through the shuffling of genes and chromosomes which goes on during the sexual cycle. Since this process provides the immediate source of variability upon which selection exerts its primary action it can be compared to the engine of the automobile. Natural selection, which directs genetic variability toward adaptation to the environment, can be compared to the driver of the vehicle." (p. 3 of Stebbins (1966) [5])

> "The basic processes of evolution are five: (1) mutation and (2) genetic recombination which are the sources of variability, but do not provide direction. . . " (p. 12 of Stebbins (1966) [5])

> "if ever it could have been thought that mutation is important in the control of evolution, it is impossible to think so now, for not only do we observe it to be so rare that it cannot compete with the forces of selection but we know this must inevitably be so." (p. 361 of Ford (1971) [12])

> "Each unitary random variation is therefore of little consequence, and may be compared to random movements of molecules within a gas or liquid. Directional movements of air or water can be produced only by forces that act at a much broader level than the movements of individual molecules, e.g., differences in air pressure, which produce wind, or differences in slope, which

> produce stream currents. In an analogous fashion, the directional force of evolution, natural selection, acts on the basis of conditions existing at the broad level of the environment as it affects populations." (p. 6 of Dobzhansky, et al., 1977 [9])

Thus, the form of the population-genetic argument about possible sources of direction in evolution is that mutation cannot be an influence because mutation rates are too small. To this, Dobzhansky, et al., add a metaphysical argument that, by analogy with statistical physics, invokes an emergent "level" of population genetics at which selection is alleged to be influential but individual variations are not.

The vestiges of the OMS theory persist in the research literature and in textbooks ("shifting gene frequencies", "gene pool", mutation as a "weak force", and so on). Nevertheless, the theory seems to be largely forgotten today. Certainly, it is difficult to find confident expressions of the theory after the 1980s. One recent exception would be the view expressed by Dawkins (2007) [13] in his critical review of Behe's *The Edge of Evolution*. Behe's thesis, in effect, is that there has not been sufficient time for all the mutations needed to account for evolution. Dawkins (2007) [13] responds by attacking the premise that evolutionary rates depend on mutation rates:

> If correct, Behe's calculations would at a stroke confound generations of mathematical geneticists, who have repeatedly shown that evolutionary rates are not limited by mutation. Single-handedly, Behe is taking on Ronald Fisher, Sewall Wright, J.B.S. Haldane, Theodosius Dobzhansky, Richard Lewontin, John Maynard Smith and hundreds of their talented co-workers and intellectual descendants. Notwithstanding the inconvenient existence of dogs, cabbages and pouter pigeons, the entire corpus of mathematical genetics, from 1930 to today, is flat wrong. Michael Behe, the disowned biochemist of Lehigh University, is the only one who has done his sums right. You think? The best way to find out is for Behe to submit a mathematical paper to The Journal of Theoretical Biology, say, or The American Naturalist, whose editors would send it to qualified referees."

The statement becomes more accurate if one interprets the word "today" in "the entire corpus ... from 1930 to today" to mean some time just prior to 1969, when origin-fixation models emerged. A still more accurate statement would be that Fisher, Wright, and Haldane *argued* that evolution is not limited by mutation, making reference to both theoretical and empirical results, on the grounds that a mutation-limited mechanism would be too slow (see p. 231 of [14]). This assumption was followed for decades and was a foundation of the OMS.

Contemporary researchers frequently contradict the theory, illustrated in the statements quoted above, specifying a passive material role for mutation in evolution. It is interesting to note that these contemporary statements are often heterogeneous, and some are confusing hybrids, e.g., stating that mutation is a "driving force" and yet also the "ultimate" source of variation— which is the preferred OMS way to signify that mutation is not a driver at all, not the proximate source of variation (which is recombination), but merely the ultimate source of variation in the distant past.

"Adaptation proceeds through the selection of mutations" Jacquier, et al. (2013) [15]

"Mutation is one of the most fundamental processes in biology. It is the ultimate source of genetic variation and one of the driving forces of evolution." Schaibley, et al. (2013) [16]

"Mutation is the engine that drives evolution and adaptation forward in that it generates the variation on which natural selection acts " Hershberg, et al. (2010) [17]

"Mutations are the main sources of evolutionary novelty, and as such constitute a key driving force in evolution" Franke, et al. (2011) [18]

Other statements, such as the following, emphasize mutation as a determinant of evolutionary rate. The statements by Bloom and by Acevedo, et al. refer indirectly to origin-fixation dynamics.

"The rate of germ line mutation is the ultimate parameter governing the amount of genetic diversity within populations and the divergence between species." Mugal and Ellegren (2011) [19]

"actual sequence evolution is governed by the rates at which mutations arise and the selection that subsequently acts on them (Halpern and Bruno 1998; Thorne et al. 2007)" Bloom (2014) [20]

"mutation rates are a key determinant of the rate of evolution" Lang, et al. (2008) [21]

"Mutation rates are central to evolution, as the rate of evolution is determined by the rate at which mutations are introduced into the population" Acevedo, et al. (2014) [22], citing Orr (2000) and Kimura (1983)

One sometimes finds statements that explicitly contrast a view based on "standing variation" and one based on "new mutations" ("mutation-limited"). The OMS assumes the former, whereas contemporary scientists debate the relative importance of these two regimes. For instance, in one statement below, Houle (1998) [23] refers to adaptation from standing variation as merely "a popular model." In other statements quoted below, the authors present the "new mutations" view as the "traditional" view or "ruling paradigm", and claim that the other view has been neglected. Note that, in some parts of the literature, there is a similar (but not precisely identical) distinction between "soft sweeps" and "hard sweeps".

"On the one hand, a popular model of adaptation assumes that the standing variance in a population is the principal source of the response to selection (e.g., Lande 1979)." Houle (1998) [23]

"We have little information about the relative importance of these two sources of beneficial alleles after a change of environment" Barrett and Schluter (2008) [24]

"Historically, population geneticists have focused attention on the hard-sweep model of adaptation in which a de novo beneficial mutation arises and rapidly

fixes in a population. Recently more attention has been given to soft-sweep models, in which alleles that were previously neutral, or nearly so, drift until such a time as the environment shifts and their selection coefficient changes to become beneficial. It remains an active and difficult problem, however, to tease apart the telltale signatures of hard vs. soft sweeps in genomic polymorphism data. " Schrider, et al. (2015) [25]

> "On the other hand, in the molecular literature on the adaptive process and on selective sweeps adaptation from a single new mutation is clearly the ruling paradigm (e.g., Maynard Smith and Haigh 1974; Kaplan et al 1989; Barton 1998; Kim and Stephan 2002). In conspicuous neglect of the quantitative genetic view, the standing genetic variation as a source for adaptive substitutions is generally ignored, with only few recent exceptions (Orr and Betancourt 2001; Innan and Kim 2004)." Hermisson and Pennings (2005) [26]

> "Most of the current theory on the genetics of adaptations assumes that adaptation occurs exclusively from new mutations rather than from standing variation" Barrett and Schluter (2008), citing Orr's work [24]

> "Models of adaptive evolution have traditionally assumed adaptation from de novo mutations," Radwan and Babik (2012) [27], citing Orr's work.

Thus, mutationist thinking has become sufficiently familiar in some contexts that researchers invoke it as a norm.

Finally, it is of interest to note that, as conceptions of evolution have shifted to include the previously heterodox idea that evolutionary change depends for its timing or character on individual mutations, this way of thinking has been projected backwards onto progenitors who rejected it, as when Dean (2012) [28] refers to "random mutation" and "the sieve of natural selection" as "basic Darwinian principles", rather than as basic Mendelian principles: a main axis of differentiation between the Mendelian view and the Modern Synthesis is whether selection sometimes acts as a sieve, i.e., according to the probability of fixation.

Likewise, Futuyma (2010) [29], in his belated partial recognition of mutation-limited dynamics, transforms the historic clash with modern neo-Darwinism into something quite different, claiming that the "Modern Synthesis" has been "modestly revised, if only by shifts in emphasis and appreciation of the significance of previously acknowledged phenomena and processes whose importance had not been widely recognized". For Mayr, Stebbins, Dobzhansky, and others, pushing mutation into the background and casting it as merely the ultimate source of raw materials, rather than allowing mutation-driven dynamics, was a critical step in justifying a Darwinian view and rejecting the preceding Mendelian view, e.g., as Mayr (1963) states

> "*Mutation as an evolutionary force.* In the early days of genetics it was believed that evolutionary trends are directed by mutation, or, as Dobzhansky (1959) recently phrased this view, 'that evolution is due to occasional lucky mutants which happen to be useful rather than harmful.' In contrast, it is held by contemporary geneticists that mutation pressure as such is of small immediate evolutionary consequence in sexual organisms, in view of the relatively far

> greater contribution of recombination and gene flow to the production of new genotypes and of the overwhelming role of selection in determining the change in the genetic composition of populations from generation to generation." (p. 101 of [2])

Mayr's conception of mutation-driven evolution may be confused, but it is relatively clear what he means to exclude. Yet, in Futuyma's conception [29], this difference is transformed into a mere shift in emphasis *within* the Modern Synthesis.

As a final example of the unconscious normalization of mutationist thinking, consider the treatment of "Fisher's geometric model" in the contemporary literature of theoretical evolutionary genetics. Fisher invoked a geometric model to determine, given a range of effect-sizes of heritable differences, from the smallest to the largest, which size is most likely to be beneficial. The answer, which is explained in various sources (e.g., [30]), is that the chance of a beneficial effect is a monotonically decreasing function of effect-size: thus, the smallest possible effects have the greatest chance of being beneficial. Fisher concluded from this that the smallest changes are the most likely in evolution, i.e., adaptation will occur gradually, by infinitesimals. A half-century later, Kimura invoked Fisher's geometric model, yet appealed to the probability of fixation of a new mutation, which *increases* with effect-size: the combination of the two factors leads to the conclusion that effects of an *intermediate* size are the most likely, because they will have the highest joint probability of being beneficial and being fixed.

That is, Kimura took Fisher's geometric model and embedded it within an origin-fixation context, fundamentally changing the result. The radical nature of Kimura's move goes unremarked in the literature of theoretical evolutionary genetics, where "Fisher's model" frequently refers to a model that assimilates Kimura's innovations (e.g., [31–33]). Typically this is an error of omission: using Fisher's name for a model without explaining that it violates his most basic beliefs about how evolution works. However, some authors are explicit in projecting Kimura's mutationist thinking backwards onto Fisher, e.g., stating that "Fisher erred here and his conclusion (although not his calculation) was flawed. Unfortunately, his error was only detected half a century later, by Motoo Kimura" [33], or stating that "an adaptive substitution in Fisher's model (as in reality) involves a 2-step process" [30].

To understand why Fisher's conclusion is not an error, recall the OMS theory that equates "evolution" with shifting gene frequencies in the gene pool. The alleles have initial frequencies determined by historical selection under the previously prevailing environment. The process of "evolution" begins when the environment changes, and selection starts to shift the frequencies to a new optimum. Most of them will simply shift up or down partially depending on whether their initial effect is beneficial or deleterious. Any unconditionally deleterious alleles will fall to their deterministic mutation-selection balance frequencies; any unconditionally beneficial ones will go to fixation deterministically. The probability of fixation for a new mutation is not relevant to the expected outcome of this process. Instead, all one needs to know is the chance of being beneficial as a function of effect-size, and Fisher's original geometric argument answers this question. Fisher's argument is complete, given his theory that evolution can be adequately understood as a deterministic process of shifting gene frequencies.

References

1. Stoltzfus, A., Cable, K.: Mendelian-Mutationism: The Forgotten Evolutionary Synthesis. J Hist Biol **47**(4), 501–46 (2014). doi:10.1007/s10739-014-9383-2
2. Mayr, E.: Animal Species and Evolution, p. 797. Harvard University Press, Cambridge, Massachusetts (1963)
3. Mayr, E.: Darwin and the Evolutionary Theory in Biology, pp. 1–10. Anthropological Society, Washington, DC (1959)
4. Mayr, E.: In: Campbell, J.H., Schopf, J.W. (eds.) The Resistance to Darwinism and the Misconceptions on which it was Based, pp. 35–46. Jones and Bartlett, Inc., London (1994)
5. Stebbins, G.L.: Processes of Organic Evolution, p. 191. Prentice Hall, Englewood Cliffs, NJ (1966)
6. Stebbins, G.L.: Darwin to DNA, Molecules to Humanity, p. 491. W.H. Freeman and Company, San Francisco (1982)
7. Dobzhansky, T.: Evolution, Genetics and Man. Wiley and Sons, Inc., New York (1955)
8. Dobzhansky, T.: In: Ayala, F., Dobzhansky, T. (eds.) Chance and Creativity in Evolution, pp. 307–338. University of California Press, Berkeley and Los Angeles (1974)
9. Dobzhansky, T., Ayala, F.J., Stebbins, G.L., Valentine, J.W.: Evolution, p. 571. W.H. Freeman, ??? (1977)
10. Yampolsky, L.Y., Stoltzfus, A.: Bias in the introduction of variation as an orienting factor in evolution. Evol Dev **3**(2), 73–83 (2001)
11. Huxley, J.S.: Evolution: The Modern Synthesis. George Allen and Unwin, London (1942)
12. Ford, E.B.: Ecological Genetics, 3rd edn., p. 410. Chapman and Hall, London (1971)
13. Dawkins, R.: Review: The Edge of Evolution (2007). http://www.iht.com/articles/2007/06/29/arts/idbriefs30A.php?page=2
14. McCandlish, D.M., Stoltzfus, A.: Modeling evolution using the probability of fixation: history and implications. Quarterly Review of Biology **89**(3), 225–252 (2014)
15. Jacquier, H., Birgy, A., Le Nagard, H., Mechulam, Y., Schmitt, E., Glodt, J., Bercot, B., Petit, E., Poulain, J., Barnaud, G., Gros, P.A., Tenaillon, O.: Capturing the mutational landscape of the beta-lactamase TEM-1. Proc Natl Acad Sci U S A **110**(32), 13067–72 (2013). doi:10.1073/pnas.1215206110
16. Schaibley, V.M., Zawistowski, M., Wegmann, D., Ehm, M.G., Nelson, M.R., St Jean, P.L., Abecasis, G.R., Novembre, J., Zollner, S., Li, J.Z.: The influence of genomic context on mutation patterns in the human genome inferred from rare variants. Genome Res **23**(12), 1974–84 (2013). doi:10.1101/gr.154971.113
17. Hershberg, R., Petrov, D.A.: Evidence that mutation is universally biased towards AT in bacteria. PLoS Genet **6**(9), 1001115 (2010). doi:10.1371/journal.pgen.1001115
18. Franke, J., Klozer, A., de Visser, J.A., Krug, J.: Evolutionary accessibility of mutational pathways. PLoS computational biology **7**(8), 1002134 (2011). doi:10.1371/journal.pcbi.1002134
19. Mugal, C.F., Ellegren, H.: Substitution rate variation at human CpG sites correlates with non-CpG divergence, methylation level and GC content. Genome Biol **12**(6), 58 (2011). doi:10.1186/gb-2011-12-6-r58
20. Bloom, J.D.: An experimentally determined evolutionary model dramatically improves phylogenetic fit. Mol Biol Evol **31**(8), 1956–78 (2014). doi:10.1093/molbev/msu173
21. Lang, G.I., Murray, A.W.: Estimating the per-base-pair mutation rate in the yeast Saccharomyces cerevisiae. Genetics **178**(1), 67–82 (2008). doi:10.1534/genetics.107.071506
22. Acevedo, A., Brodsky, L., Andino, R.: Mutational and fitness landscapes of an RNA virus revealed through population sequencing. Nature **505**(7485), 686–90 (2014). doi:10.1038/nature12861
23. Houle, D.: How should we explain variation in the genetic variance of traits? Genetica **102-103**(1-6), 241–53 (1998)
24. Barrett, R.D., Schluter, D.: Adaptation from standing genetic variation. Trends Ecol Evol **23**(1), 38–44 (2008)
25. Schrider, D.R., Mendes, F.K., Hahn, M.W., Kern, A.D.: Soft shoulders ahead: spurious signatures of soft and partial selective sweeps result from linked hard sweeps. Genetics **200**(1), 267–84 (2015). doi:10.1534/genetics.115.174912
26. Hermisson, J., Pennings, P.S.: Soft sweeps: molecular population genetics of adaptation from standing genetic variation. Genetics **169**(4), 2335–52 (2005)
27. Radwan, J., Babik, W.: The genomics of adaptation. Proceedings. Biological sciences / The Royal Society **279**(1749), 5024–8 (2012). doi:10.1098/rspb.2012.2322
28. Dean, A.M.: Evolution: a View from the 21st Century. Microbe Magazine (March) (2012)
29. Futuyma, D.J.: Evolutionary constraint and ecological consequences. Evolution **64**(7), 1865–84 (2010). doi:10.1111/j.1558-5646.2010.00960.x
30. Orr, H.A.: Theories of adaptation: what they do and don't say. Genetica **123**(1-2), 3–13 (2005)
31. Matuszewski, S., Hermisson, J., Kopp, M.: Fisher's geometric model with a moving optimum. Evolution **68**(9), 2571–88 (2014). doi:10.1111/evo.12465
32. Blanquart, F., Achaz, G., Bataillon, T., Tenaillon, O.: Properties of selected mutations and genotypic landscapes under Fisher's geometric model. Evolution **68**(12), 3537–54 (2014). doi:10.1111/evo.12545
33. Orr, H.A.: The genetic theory of adaptation: a brief history. Nat Rev Genet **6**(2), 119–27 (2005)
